# Experimental evaluation of the effects of commercial additive (plant extracts) as an alternative to growth-promoting antibiotics in broiler chickens

**DOI:** 10.14202/vetworld.2025.636-645

**Published:** 2025-03-18

**Authors:** María Julieta Luna, Maite Corti Isgro, Lorenzo Alejandro Rosales Cavaglieri, María Valeria Coniglio, María Eugenia Ortiz, Lilia René Cavaglieri, Julián Parada, Alejandra Paola Magnoli

**Affiliations:** 1 Department of Animal Production, National University of Río Cuarto, Córdoba, Argentina; 2 Fellow of National Council for Scientific and Technological Research, Buenos Aires, Argentina; 3 Department of Animal Pathology, National University of Río Cuarto, Córdoba, Argentina; 4 Department of Microbiology and Immunology, National University of Río Cuarto, Córdoba, Argentina; 5 Member of National Council for Scientific and Technological Research, Buenos Aires, Argentina

**Keywords:** antibiotic alternatives, broiler chickens, carvacrol, cinnamaldehyde, gut health, phytogenic feed additives

## Abstract

**Background and Aim:**

The increasing prevalence of antibiotic-resistant pathogens necessitates the exploration of alternatives to growth-promoting antibiotics (GPAs) in poultry production. This study evaluated a commercial additive containing plant extracts (carvacrol and cinnamaldehyde) as a potential replacement for GPAs in broiler chickens, focusing on productive parameters, cecal microbiota composition, cecal volatile fatty acid (VFA) concentration, and small intestinal histomorphology.

**Materials and Methods:**

The study involved 100 one-day-old Cobb 500 broiler chickens, divided into two treatment groups: Group 1 (control) received a basal diet (BD) with avilamycin (100 g/T), and Group 2 received a BD with a phytogenic feed additive (PFA) containing 10% carvacrol and 10% cinnamaldehyde (100 g/Tn). Over 42 days, the study measured total weight gain (TWG), feed intake, feed conversion ratio (FCR), carcass yield, digestive tract length, intestinal histomorphometry, VFA concentration, and cecal microbiota composition.

**Results:**

The PFA-treated group showed a 1.67% improvement in TWG and a 5.7% improvement in FCR compared to the control. The digestive tract length increased by 20 cm with PFA supplementation. While no significant differences were observed in cecal microbiota counts and VFA concentrations, a trend toward increased lactic acid bacteria and VFA levels was noted. Histomorphological analysis indicated enhanced villus height and a higher villus height-to-crypt depth ratio in the PFA group.

**Conclusion:**

The findings suggest that carvacrol and cinnamaldehyde-based PFAs may serve as viable alternatives to GPAs, promoting growth performance and gut health in broiler chickens. Further research is needed to elucidate the underlying mechanisms of action and confirm these preliminary results in larger-scale studies.

## INTRODUCTION

For several decades, growth-promoting antibiotics (GPAs) have been incorporated into the diets of poultry, pigs, and ruminants to enhance productive performance and prevent diseases in livestock [1]. Antibiotics are used as growth promoters to minimize the incidence and severity of subclinical infections, reduce undesirable intestinal flora, and decrease the production of bacterial metabolites that negatively affect growth [2]. However, the use of antibiotics in poultry feed has raised significant health concerns for humans, primarily due to the emergence of antibiotic-resistant pathogenic bacteria and the bioaccumulation of antibiotic residues in poultry meat and eggs [3]. Antimicrobial resistance (AMR) can develop through two primary mechanisms. The first involves resistance mediated by pre-existing phenotypic traits in natural bacterial populations, while the second mechanism involves acquired resistance, which may arise through direct gene mutations or indirectly through the acquisition of resistance-encoding DNA fragments [3]. Given the growing concerns regarding antibiotic-resistant pathogens posing a public health risk, regulatory actions have been implemented to limit antibiotic use. Notably, the European Union banned GPAs in 2006 to reduce potential risks to consumer health [4]. This ban prompted the poultry industry to optimize farm management practices, enhance biosecurity, improve environmental controls, and modify poultry diet formulations [5]. However, the complete removal of antibiotic growth promoters (AGPs) could lead to an increased incidence of pathogenic infections, potentially compromising livestock health and productivity [6]. Consequently, there is a strong incentive to identify safe alternatives that not only mimic AGP-like effects, such as protecting the animal’s digestive tract from pathogens but also do not adversely affect the overall health of the animal [6]. A wide variety of feed additives are now used in poultry production as potential alternatives to AGPs. These include phytogenic feed additives (PFAs) such as essential oils, herbal extracts, and organic acids, as well as prebiotics, probiotics, and enzymes [1, 7_12]. Among these, PFAs – also referred to as phytobiotics or botanicals – have attracted considerable attention in the poultry sector over the past decade. Their gradual integration into commercial poultry diets aims to improve productive performance and promote health by controlling enteric pathogens [5]. According to European Union legislation, plant-based additives are categorized as sensory and flavoring compounds and consist primarily of plant extracts, including essential oils, oleoresins, flavonoids, and their active components [4]. Bioactive molecules present in PFAs, such as carvacrol, thymol, capsaicin, and cineole, contribute to enhanced growth performance, nutrient digestibility, and gut health in poultry [6, 13, 14]. Research by Granstad *et al*. [15] highlighted the antimicrobial properties of PFAs against pathogenic bacteria such as *Escherichia coli* and *Clostridium perfringens* in broiler chickens, effectively reducing the risk of colibacillosis and necrotic enteritis. These antimicrobial effects are attributed to the phenolic components of PFAs and their action on pathogenic cells [16]. Moreover, PFAs have been shown to improve nutrient utilization in the gastrointestinal tract by stimulating digestive secretions and enzymatic activity [17, 18]. They also positively affect the morphology of small intestinal tissues by increasing villus height, decreasing crypt depth, and enhancing goblet cell counts [19_21].

In this study, we focused on carvacrol, a phenolic compound found in oregano (*Origanum vulgare*) essen- tial oil, and cinnamaldehyde, an aromatic molecule derived from cinnamon (*Cinnamomum verum*). Both compounds exhibit proven antibacterial and antioxidant properties, making them valuable as phytobiotics in broiler nutrition [22]. While previous studies by Luna *et al*. [23] and Facchi *et al*. [24] have independently reported the benefits of carvacrol and cinnamaldehyde in improving productive parameters and gut health in broiler chickens, the combined effects of these compounds as an alternative to GPAs remain underexplored.

The use of plant-based biological additives such as carvacrol and cinnamaldehyde offers a promising strategy to enhance intestinal microbiota, boost production parameters, and reduce reliance on non-therapeutic antibiotics in poultry farming. This approach aligns with the growing emphasis on the “One Health” perspective, promoting sustainable agricultural practices that safeguard animal, human, and environmental health.

## MATERIALS AND METHODS

### Ethical approval

The study’s protocol and methodologies adhered to the guidelines set forth by the Animal Bioethics Subcommittee under the Scientific Research Ethics Committee of the National University of Río Cuarto (Approval N^o^ 383/22).

### Study period and location

The study was conducted from March to June 2023 at Experimental Unit of Animal Nutrition of the Faculty of Agronomy and Veterinary Medicine of the National University of Río Cuarto, Córdoba, Argentina. The rainy season lasts for approximately 10 months, with a sliding 31-day rainfall of at least 13 mm and an average total accumulation of 131 mm. The period of the year without rain lasts approximately 2 months, with an average total accumulation of 8 mm. The temperate season has an average daily maximum temperature of 27°C and an average minimum temperature of 19°C. The cool season has an average daily maximum temperature of <18°C and an average minimum temperature of 5°C.

### Phytogenic additive as a nutritional supplement

The novel PFA used in this study was formulated with 10% carvacrol and 10% cinnamaldehyde. It was administered in powder form at the manufacturer’s recommended dosage of 100 g/Tn (Bedson S.A., Argentina).

### GPA

The GPA avilamycin (10%) was commercially sourced (Bedson S.A.) and included in the feed at a concentration of 100 g/Tn.

### Animals and housing

One-day-old male Cobb 500 broiler chickens (initial body weight 0.0472 ± 0.020 kg, total n = 100), vaccinated against Marek’s disease, were obtained from a commercial hatchery (INDACOR SA, Córdoba, Argentina). The birds were acclimatized for 1 week before the experiment. Throughout the study, feed and water were provided *ad libitum*. The lighting regimen included continuous light during the 1^st^ week, followed by a 23-h light and 1-h dark cycle for the remainder of the 42-day experimental period. The chickens were randomly allocated to stainless steel cages and arranged into 10 replicates per group, with 5 chickens per cage (90 × 100 × 39 cm). The birds were fed a standard maize-soybean meal starter commercial diet (basal diet [BD]) (Table 1), with or without GPA (10% avilamycin), formulated according to National Research Council guidelines [25]. Experimental diets were prepared by mixing the BD with PFA additive or avilamycin using a 130 L food mixing machine equipped with a WEG electric motor (WEG, Argentina). The experimental design included two groups: Group 1 (control) received a BD with avilamycin (100 g/T), while Group 2 received a BD without avilamycin but supplemented with the PFA additive (100 g/Tn).

**Table 1:** Composition of basal diet (%, as fed basis).

Ingredient	Diet	

	Grower	Finisher
Yellow corn	62.90	67.20
Soybean oil meal	22.60	19.0
Soybean heat treated	5.50	5.0
Meat and bone meal	6.90	7.0
Vitamin and mineral mix^1^	0.15	0.15
NaCl	0.20	0.20
Oystershell	0.35	0.30
Sunflower oil	1.0	1.0
DL-Methionine	0.16	0.10
L-Lysine	0.10	-
Monensin	0.05	0.05
Total	100	100
Proximate composition (g/kg diet)		
Crude protein	20.33	18.90
Crude fat	5.47	5.53
Crude fiber	3.34	3.08
Calcium	0.97	0.95
Total phosphorus	0.59	0.57
Lysine	1.14	0.93
Methionine	0.50	0.42
Tryptophan	0.24	0.22
Metabolizable energy (Kcal/Kg)	3047	3062

1 The premix contained the following per kg of powder: calcium 10.2%, starch 0.016%, crude fiber 0.012%, vitamin A 1,600,000 IU, vitamin D3 320,000 IU, vitamin E 4,800 IU, vitamin B1 320 mg, vitamin B2 800 mg, vitamin B6 640 mg, vitamin B12 3,200 μg, vitamin K3 320 mg, pantothenic acid 1600 mg, niacin 6400 mg, biotin 24,000 μg, folic acid 160 mg, choline chloride 24,000 mg, iron 6400 mg, iodine 160 mg, copper 1600 mg, manganese 12,800 mg, zinc 9600 mg, and selenium 24 mg. BD=Basal diet

### Evaluated parameters

#### Productive parameters

The broiler chickens were weighed individually on a weekly basis and at the conclusion of the study. Daily monitoring for signs of morbidity and mortality was also conducted. Productive parameters were assessed over three intervals: Days 1–21, days 22–42, and the entire study period (days 1–42). Total weight gain (TWG, kg) was calculated per pen as the difference between the final and initial weights. Feed intake (FI, kg) was recorded for each animal, and the feed conversion ratio (FCR) was determined as the ratio of FI to TWG for each period.

#### Carcass parameters and length of the intestinal tract

At 48 days, 40 chickens (20 per group, 2 per replicate) were randomly selected and euthanized by white bloodletting, following the ethical guidelines of the regulations of the Subcommittee on Animal Bioethics under the Ethics Committee of Scientific Research, as established in Resolution 253/10 of the Superior Council of the National University of Río Cuarto. A thorough necropsy was performed to determine the weights of key carcass cuts (leg-thigh and breast) and to measure the length of the digestive tract from the pylorus (in cm).

#### Cecal microbiota count

Cecal content samples (20 per group) were collected aseptically in sterile tubes and immediately transported on ice to the Microbiology Laboratory, School of Veterinary Medicine, National University of Río Cuarto, for bacteriological analysis using the methodology described by Baroni *et al*. [26]. One gram of cecal content was serially diluted, and 10 μL of each dilution was inoculated onto De Man Rogosa Sharpe Agar (Merck, Germany) and MacConkey agar plates to quantify lactic acid bacteria and enterobacteria, respectively. Microbial counts were obtained after 24-h aerobic incubation at 37°C for enterobacteria and 48-h aerobic incubation at 37°C for lactic acid bacteria. Microbiota evaluation involved counting the colony-forming units (CFUs) per gram using methods outlined by Behnamifar *et al*. [27].

#### Determination of volatile fatty acids (VFAs)

At 21 days of the trial, 4 chickens (2 per group) were sacrificed for cecal content collection, with an additional 40 chickens (20 per group) sacrificed at 48 days. Samples were frozen at –20°C until VFA concentrations were analyzed. The VFA analysis followed the method of Park *et al*. [28], with modifications. One gram of cecum was mixed with 1 mL of distilled water and centrifuged at 5,000× *g* for 20 min at 4°C. Subsequently, 200 μL of 25% (w/v) metaphosphoric acid was added, and samples were centrifuged again at 5,000× *g* for 10 min at 4°C. The supernatant was analyzed using gas chromatography to determine acetic, propionic, and butyric acid concentrations, as described by Parada *et al*. [29].

#### Histomorphology of the small intestine

Twenty duodenum samples per group were collected for histological analysis, following the methodology described by Poloni *et al*. [30]. Morphometric parameters, including villus length, width, and crypt depth, were measured according to the protocol of Nain *et al*. [31].

#### Apparent absorptive area (AAA)

The AAA of the duodenal villus was estimated using the methodology described by Magnoli *et al*. [11].

### Statistical analysis

Data were statistically analyzed using a general linear mixed model (GLMM) (version 2.03; Córdoba, Argentina). Analysis of variance (ANOVA) was performed, and mean comparisons were conducted using Fisher’s protected least significant difference (LSD) test with a significance level set at p < 0.05.

The collected data were analyzed using a GLMM with version 2.03 of the statistical software (Córdoba, Argentina). The ANOVA was performed to determine the significance of differences between the treatment groups. Productive parameters, including TWG, FI, and FCR, as well as histomorphological measurements and microbiota counts, were treated as dependent variables, while treatment groups (GPA vs. PFA) were considered fixed factors.

For the comparison of means, Fisher’s protected LSD test was applied, with a significance level set at p < 0.05. Data normality was assessed using the Shapiro–Wilk test, and homogeneity of variances was verified with Levene’s test. Non-normally distributed data were log-transformed before analysis.

Results are presented as mean ± standard error of the mean. Where applicable, pairwise comparisons were performed, and significant differences between treatment groups were denoted by distinct superscripts (e.g., a, b).

## RESULTS

### Productive parameters

Mortality was 10% for the control group (5/50) and 5% for the PFA additive group (1/50) (p ≤ 0.05) for the period assayed. No statistically significant differences in TWG were found during the evaluated periods (p ≥ 0.05) (Table 2). The addition of the additive without GPA showed similar behavior to the control with GPA in terms of TWG, with values 6.56% and 1.66% higher than the control group during the 1–21 day and 1–42 day periods, respectively. No statistically significant differences were found in FI (p ≥ 0.05) between the groups throughout the experimental periods. However, during 1–42-day periods, the FI of the PFA additive group was lower by 3.93% compared to control with GPA (Table 2). No statistically significant differences were found in FCR (p ≥ 0.05) between the tested groups in the 1–22-day and 22–42-day experimental periods (Table 2). However, supplementation with the PFA additive resulted in a lower FCR compared with the control group during the same period. On the other hand, supplementation with the PFA resulted in a 5.73% lower FCR compared with the GPA-fed broiler chickens during the 1–42-day period (p ≤ 0.05). Table 2 shows the breast and leg-thigh weights of broiler chickens from both treatment groups during the 1–42-day period. The poultry breast and leg-thigh weights did not differ significantly between groups (p ≥ 0.05).

**Table 2:** Effects of different treatments on production parameters and digestive tract length in broiler chickens at 1–42 days of the assay and weight of economically important cuts from broiler chickens.

Parameters	1–21 days				22–42 days				1–42 days			
		
	G1	G2	p-value	SEM	G1	G2	p-value	SEM	G1	G2	p-value	SEM
TWG (kg)	1.253	1.341	0.13	0.04	1.786	1.749	0.53	0.04	3.039	3.090	0.58	0.07
FI (kg)	1.991	2.065	0.40	0.06	3.740	3.508	0.09	0.09	5.927	5.694	0.16	0.32
FCR	1.592	1.542	0.19	0.03	2.094	2.005	0.08	0.03	1.953^a^	1.841^b^	0.04	0.04
Poultry breast (g)									855.4	902.4	0.28	30.85
Leg-thigh (g)									674.3	660.5	0.66	22.84
The length of the digestive tract (cm)	148	150	0.94	0.14					158^b^	178^a^	0.003	0.04

^a,b^Values in columns with no common superscripts are significantly different (p < 0.05) according to Fisher’s protected least significant test (LSD test). G1=Basal diet with avilamycin 100 g/T, G2=Basal diet without avilamycin with PFA additive 100 g/Tn, TWG=Total weight gain per pen, FI=Feed intake per animal, FCR=Feed conversion ratio (kg FI/kg TWG), BD=Basal diet, SEM=Standard error of means, PFA=Phytogenic feed additive

The digestive tract lengths of broiler chickens from different treatment groups during the 1–21-day and 1–42-day periods are shown in Table 2. Results did not show significant differences between groups in the 1–21-day period (p ≥ 0.05). On the other hand, during the 1–42-day period, supplementation with the PFA additive significantly increased the length of the digestive tract (20 cm longer) compared with the control group (p ≤ 0.05).

### Cecal microbiota count

The counts of lactic acid bacteria and enteric bacteria at 21 and 42 days are shown in Table 3 (CFU/g wet cecal digesta). The counts of lactic acid bacteria and enteric bacteria did not show significant differences with the supplementation of the PFA additive compared with the control group in either of the tested periods (p ≥ 0.05). In contrast, PFA supplementation decreased the number of cecal enteric bacteria and increased the number of cecal lactic acid bacteria in either of the tested periods.

**Table 3:** Effects of PFA supplementation on the counts of lactic acid bacteria and enteric bacteria (CFU/g) and VFA concentrations (mM/g) in ceca of the broiler chickens at 21 and 42 days of the assay*.

Parameters	1–21 days				1–42 days			
	
	G1	G2	p-value	SEM	G1	G2	p-value	SEM
Enteric bacteria	6.60 × 10^8^	4.55 × 10^8^	0.64	2.73 × 10^8^	6.40 × 10^8^	1.70 × 10^7^	0.0^9^	2.5 × 10^8^
Lactic acid bacteria	2.89 × 10^8^	1.93 × 10^9^	0.43	1.97 × 10	5.10 × 10^7^	6.89 × 108	0.0^7^	1.66 × 10^8^
VFAs (mM/g)								
Acetic	5.82	14.02	0.27	3.33	7.90	10.37	0.40	1.53
Propionic	0.69	1.25	0.07	3.33	1.14	1.29	0.80	0.23
Butyric	1.53	2.02	0.47	0.64	1.89	2.92	0.49	0.60

p-values >0.05 do not show significant differences.

* n = 20 cecum/treatment. G1=Basal diet with avilamycin 100 g/T, G2=Basal diet without avilamycin with PFA additive 100 g/Tn, PFA=Phytogenic feed additive, SEM=Standard error of the mean, CFU=Colony-forming units, VFA=Volatile fatty acid

### Determination of VFAs

Table 3 shows the concentrations of VFAs (mM/g) of the cecal contents of broiler chickens at 1–21 days and 1–42 days. Acetic acid was the predominant VFA in both experimental periods, followed by butyric and propionic acids. The concentration of VFA did not differ significantly between treatment groups (p ≥ 0.05) in either experimental period. In contrast, broiler chickens supplemented with the PFA exhibited the highest VFA values in both periods.

### Histomorphology of the small intestine

The mean values of duodenal VH, VW, CD, AAA, and VH/CD are presented in Table 4. The VH and VH/CD of animals supplemented with the PFA additive were significantly higher than those of the control group (p ≤ 0.05). VW and AAA were not affected by the dietary inclusion of PFA. However, AAA showed positive results in the PFA additive group compared with the control.

**Table 4:** Length, width, crypt depth, apparent absorptive area, and height/crypt depth ratio of the small intestine (duodenum) of broiler chickens supplemented with PFA additive APG during 1–42 days.

Parameters	Groups			

	G1	G2	p-value	SEM
VH (µm)	1,161.36^b^	1,434.63^a^	0.0004	51.74
VW (µm)	152.34	143.26	0.3860	7.35
CD (µm)	248.89^a^	172.42^b^	<0.0001	11.23
AAA (µm^2^)	554,408.35	639,669.79	0.09	35,134.58
VH/CD	4.97^b^	9.59^a^	<0.0001	0.64

^a,b^Values in columns with no common superscripts are significantly different (p ≤ 0.05) according to Fisher’s protected least significance test (LSD test). *n = 40 broiler chickens, 20 per treatment. VH=Villus height, VW=Villus width, CD=Crypt depth, AAA=Apparent absorptive area, VH/CD=Villus height/crypt depth ratio, SEM=Standard error of the mean, G1=Basal diet with avilamycin 100g/T, G2=Basal diet without avilamycin with PFA additive 100 g/Tn

**Figure 1:**
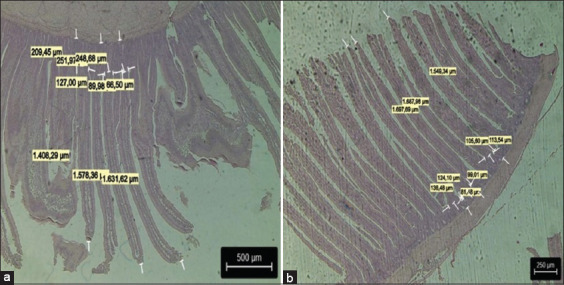
Photomicrographs of hematoxylin and eosin-stained chicken duodenum sections ×20. (a) G1: Basal diet (BD) with avilamycin 100 g/T; (b) G2: BD without avilamycin with phytogenic feed additive 100 g/Tn. (a) Scale bars: 500 and (b) scale bar 250 μm.

Figure 1 shows the effects of the dietary inclusion of PFA on histomorphological parameters. The duodenum shows proper villi development with no signs of inflammation or atrophy. The lieberkuhn glands showed normal development and a high proportion of villus height in all treatment groups.

## DISCUSSION

Phytogenic additives support animal growth through various mechanisms, including modulating the intestinal microbiota, enhancing digestibility and nutrient absorption, maintaining gastrointestinal tract integrity, and exhibiting antioxidant properties [12].

In this study, carvacrol and cinnamaldehyde were used as PFAs to assess their impact on productive performance, microbiota composition, cecal VFA concentrations, and small intestinal histomorphology as alternatives to GPAs in broiler chicken production.

Previous research by Hussein *et al*. [32] has indicated that the productive performance of broiler chickens fed with feed additives is often comparable to that achieved using GPAs. However, some studies have suggested that feed additives may not offer the same benefits as GPAs [33, 34]. Despite this, achieving comparable results to the control treatment with GPAs can still justify replacing antibiotics as growth promoters, given the harmful effects and AMR associated with antibiotic use. In this study, the inclusion of PFA containing carvacrol and cinnamaldehyde (100 g/Tn) demonstrated effects similar to those of the control with GPA on productive parameters, including reduced FCR and FI, alongside increased TWG. These improvements may be attributed to the PFA’s antimicrobial and antioxidant activities, which enhance gut health and stimulate digestive enzymes, promoting better nutrient digestion and growth [35, 36].

Our findings align partially with earlier studies that reported improved FCR in broiler chickens using PFAs such as lavender (*Lavandula angustifolia*) essence at concentrations between 100 and 800 mg/kg for 42 days [37]. Likewise, a study demonstrated that incorporating capsicum and other spice extracts (e.g., black pepper and ginger) at 250 parts per million for 21 days enhanced growth performance at 7 days of age and positively influenced nutrient digestibility and antioxidant response [38]. Falaki *et al*. [39] found that replacing antibiotics with 150 mg/kg of *Carum copticum* essential oil improved productive performance and decreased undesirable intestinal bacteria in broiler chickens. Conversely, Cerisuelo *et al*. [40] did not observe significant changes in productive parameters with varying doses of a commercial blend of cinnamaldehyde and thymol (0, 50, and 100 mg/kg) combined with 1 g/kg of sodium butyrate.

In this study, PFA supplementation did not significantly affect poultry breast and leg-thigh weights, although poultry breast weights exhibited behavior similar to that of the control group (GPA). These results contrast with Williams *et al*. [41], who reported increased breast weight following supplementation with a 1% mixture of Ethiopian pepper and clove in broiler chickens. In addition, Khatun *et al*. [42] observed enhanced breast meat yield with the inclusion of a phytobiotic natural extract from *Macleaya cordata* (0.20 g/kg feed) in broilers.

Maintaining a healthy intestinal microbiota and gut integrity is crucial for avian health and poultry productivity. PFAs may contribute to improved chicken health by promoting a balanced intestinal ecosystem, as highlighted by various studies describing the antimicrobial properties of PFAs [12]. However, the precise mechanism by which PFAs support the proliferation of beneficial bacteria, such as *Lactobacillus* spp., remains unclear [43]. Abd El-Hack *et al*. [44] proposed that the phenolic structures of PFAs might interact with bacterial cytoplasmic membranes, altering their integrity and functionality, which could help control the growth of pathogenic bacteria in the gut.

In this study, while carvacrol and cinnamaldehyde supplementation did not lead to statistically significant changes in cecal counts of enterobacteria and *Lactobacillus* spp., a trend toward increased lactic acid bacteria populations was observed. In contrast, Hashemipour *et al*. [45] reported a rise in the *Lactobacillus* population when broiler chickens aged 0–24 days were fed a phytogenic product containing thymol and carvacrol. Similarly, Murugesan *et al*. [20] observed a reduction in cecal coliforms and an increase in *Lactobacillus* spp. when a commercial phytogenic product (150 mg/kg) was included in the broiler diet.

The current study also showed that carvacrol and cinnamaldehyde supplementation tended to increase villus height, the villus height-to-crypt depth (VH/CD) ratio, and the absorptive area in the duodenum. Both PFAs and GPAs generally promote greater villus height in the small intestine, enhancing the absorptive area and improving digestion efficiency. These findings are consistent with Murugesan *et al*. [20], who reported significantly increased villus height and reduced crypt depth in PFA-fed birds compared to GPA-fed chickens.

Du *et al*. [46] similarly demonstrated that essential oils containing 25% thymol and 25% carvacrol improved the VH/CD ratio. Pham *et al*. [47] also observed increased villus height and VH/CD ratios with a blend of encapsulated essential oils and organic acids (BLJ) containing 4% thyme, 4% carvacrol, 0.5% hexanoic acid, 3.5% benzoic acid, and 0.5% butyric acid, administered at 0 and 500 mg/kg in the diet. Conversely, Feng *et al*. [48] did not find significant histomorphometric changes with the addition of oregano essential oil (containing carvacrol and thymol) in laying hens. However, these authors noted a linear increase in villus height and crypt depth with higher EO supplementation, suggesting an enhanced AAA.

In this study, broiler chickens fed a diet with carvacrol and cinnamaldehyde also exhibited significantly longer digestive tracts. These results align with Camay *et al*. [49], who reported increased digestive tract length in broilers supplemented with curcumin (2.5 g, 5.0 g, 7.5 g, and 10 g). Similarly, adding 500 mg of curcumin to the diet of rats led to greater small intestinal length in all experimental groups compared to controls [50]. A longer intestine may improve fluid and nutrient absorption efficiency and aid in the effective breakdown of ingested feed.

In addition, our results indicated that dietary supplementation with carvacrol and cinnamaldehyde elevated the levels of cecal VFAs such as acetic, butyric, and propionic acids. Ma *et al*. [51] found similar increases in acetate, butyrate, and isobutyrate concentrations in broilers fed a mixed organic acid blend for 42 days. Aljumaah *et al*. [52] also showed that combining short- and medium-chain organic acids with β1-4 mannobiose increased VFA levels in cecal contents. VFA production during microbial fermentation depends on the intestinal environment and microbiota, contributing to anti-inflammatory and immune-enhancing effects by regulating epithelial barrier function [51].

Variations in dosage, application methods, diet composition, and management conditions may result in differences in outcomes across studies on PFA supplementation. Careful evaluation and monitoring of non-nutritional additives are essential, emphasizing animal welfare and food safety. Future research should focus on developing new alternatives to promote healthy and sustainable poultry growth. Larger studies are needed to assess microbial diversity, elucidate the mechanisms of improved production performance, and evaluate the cost-effectiveness of additive inclusion.

## CONCLUSION

This study demonstrated that replacing GPAs with a PFA containing carvacrol and cinnamaldehyde significantly enhances the productive performance and gut health of broiler chickens. The experimental results showed a 1.67% improvement in TWG and a 5.7% enhancement in FCR in the PFA-treated group compared to the control. In addition, PFA supplementation resulted in a notable increase of 20 cm in digestive tract length and positively influenced intestinal histomorphology by improving villus height and the VH/CD ratio. Although there were no statistically significant differences in the counts of cecal microbiota and VFA concentrations, trends toward increased lactic acid bacteria and VFA levels were observed, suggesting potential gut health benefits.

The strength of this study lies in its comprehensive evaluation of productive parameters, gut microbiota composition, and small intestinal morphology, providing a holistic view of the additive’s efficacy as an alternative to GPAs. The use of a well-structured experimental design with randomized treatment groups and rigorous statistical analysis contributes to the robustness of the findings.

However, the study also has certain limitations. The sample size was relatively small (100 broiler chickens), which may limit the generalizability of the results to larger commercial poultry settings. In addition, while preliminary findings are promising, the study did not fully elucidate the underlying mechanisms by which carvacrol and cinnamaldehyde exert their beneficial effects on gut health and growth performance. The absence of significant changes in cecal microbiota and VFA concentrations, despite observable trends, warrants further investigation with advanced microbiological and molecular techniques.

Future research should focus on larger-scale trials to validate these findings under commercial poultry production conditions. Investigating the molecular and biochemical pathways through which PFA influences gut health and productivity could offer deeper insights into its mode of action. In addition, exploring different dosages, combinations with other natural additives, and long-term impacts on poultry health and product quality will further support the development of sustainable and antibiotic-free livestock farming practices.

## AUTHORS’ CONTRIBUTIONS

MJL: Data curation, formal analysis, investigation and methodology, and writing–original draft, review, and editing of the manuscript. MCI and LARC: Data curation, investigation, and methodology. MVC: Formal analysis. MEO: Formal analysis. LRC: Conceptualization and review and editing of the manuscript. JP: Conceptualization and writing–original draft, review, and editing of the manuscript. APM: Project administration, supervision, and writing–original draft, review, and editing of the manuscript. All authors have read and approved the final manuscript.
